# Human Mesenchymal Stem Cell-Derived Exosomal microRNA-143 Promotes Apoptosis and Suppresses Cell Growth in Pancreatic Cancer via Target Gene Regulation

**DOI:** 10.3389/fgene.2021.581694

**Published:** 2021-02-12

**Authors:** Bingyi Wang, Yan Xu, Yuhua Wei, Lixin Lv, Nanbin Liu, Rui Lin, Xiuyan Wang, Baomin Shi

**Affiliations:** ^1^Department of General Surgery, Tongren Hospital, Shanghai Jiao Tong University School of Medicine, Shanghai, China; ^2^Department of General Surgery, Tongji Hospital, Tongji University School of Medicine, Shanghai, China; ^3^Department of Hematology, Tongji Hospital, Tongji University School of Medicine, Shanghai, China; ^4^Department of Ultrasonography, Tongji Hospital, Tongji University School of Medicine, Shanghai, China

**Keywords:** pancreatic cancer, mesenchymal stem cells, exosomes, microRNAs, long non-coding RNAs

## Abstract

**Background:**

This study aimed to explore the regulatory mechanism of hsa-miR-143-3p and lncRNA RP11-363N22.3–functioning upstream of *KRAS*–in exosomes derived from human mesenchymal stem cells (hMSCs) in pancreatic cancer.

**Methods:**

Western blotting and quantitative PCR were used to determine gene expression. *In vitro*, cell proliferation, apoptosis, and cell cycle and invasion were evaluated using CCK-8 assay, flow cytometry, and transwell assays, respectively. *In vivo*, the effect of hsa-miR143-3p was investigated using a tumorigenesis test in nude mice. The association between hsa-miR-143-3p and lncRNA RP11-363N22.3 was investigated using the dual-luciferase assay.

**Results:**

hsa-miR-143-3p expression significantly increased in hMSC exosomes than in those in human pancreatic cancer cell line (CFPAC-1) exosomes. *In vitro*, compared to the MOCK (CFPAC-1 only) group, cell proliferation and invasion were inhibited and apoptosis was induced in the inhibitor NC (CFPAC-1 + MSC-hsa-miR-3p inhibitor NC) group, while these changes were reversed in the inhibitor (CFPAC-1 + MSC-hsa-miR-3p inhibitor) group. The expression of lncRNA RP11-363N22.3 and genes related to miR-143 significantly decreased in the inhibitor NC group compared to the MOCK group, and increased in the inhibitor group compared to inhibitor NC group. A targeted combinatorial effect was observed between lncRNA RP11-363N22.3 and hsa-miR-143-3p. *In vivo*, the tumor volume of the mimics (CFPAC-1 + MSC-hsa-miR-143-3p mimics) group was smaller than that of the mimics NC (CFPAC-1 + MSC-hsa-miR-143-3p mimics NC) and MOCK groups. H&E staining showed that there were no obvious pathological changes in MOCK and mimic NC groups, while cell necrosis was seen in some regions in mimic groups.

**Conclusion:**

hsa-miR-143-3p may promote apoptosis and suppress cell growth and invasion in pancreatic cancer.

## Introduction

Pancreatic cancer has extremely poor prognosis and is the fifth leading cause of cancer-related deaths (Reis et al., 2017). The common risk factors include smoking, specific inherited genetic syndromes, and a family history of pancreatic cancer (Thomas, 2017). The mortality rate of this devastating disease has barely improved over the past decade (Kang et al., 2014). The 5-year survival rate of pancreatic cancer is only 3–5%, and its incidence is increasing in several countries (Timofte et al., 2014). In recent decades, chemotherapy has emerged as one of the primary therapeutic modalities for pancreatic cancer, although the low curative rate limits its clinical application (Meng et al., 2017). Therefore, extensive studies are necessary to discover more effective treatments for pancreatic cancer.

Exosomes are nanoscale particles secreted by various types of cells that transport signaling molecules, such as non-coding RNAs, mRNAs, and proteins, between cells (Schneider and Simons, 2013). It has been reported that exosomes are associated with changes in tumor behavior, such as growth and metastasis (Taverna et al., 2012). Mesenchymal stem cells (MSCs), a heterogeneous population of cells, play a crucial role in cancer (Gu et al., 2016). Owing to their huge capacity to produce exosomes and due to their additional functionalities, such as immunosuppression, immune regulation, and antitumor effects, MSCs are considered an ideal source of exosomes (Yeo et al., 2013). MSCs have been widely investigated in clinical studies as they have various physiological functions, such as multilineage differentiation, promoting tissue-repair, mediating inflammatory and immune systems, and so on. The beneficial properties of MSCs are partly relying on their ability to home to injured tissues, and they could differentiate and replace injured cells. Nevertheless, these behaviors were found to be low and transient at injury sites. Studies showed the therapeutic effects of MSCs primarily in a paracrine manner (Katsha et al., 2011). Exosomes take part in cellular communication, and they are considered as the paracrine effectors of MSCs. It had been demonstrated that exosomes that derived from MSCs showed similar functions to MSCs (Yu et al., 2014; Yaghoubi et al., 2019). *KRAS* has been regarded as an important predictor of pancreatic cancer progression and understanding its role may provide valuable information that might help in the development of effective pancreatic cancer therapies. As a key modulator of pancreatic cancer, *KRAS* mutation is a potential therapeutic target (Guerra and Barbacid, 2013). It has been reported that oncogenic *KRAS* mutations, and subsequent activation of the downstream effectors, such as Erk, Akt, and MEK, can result in the modulation of pancreatic cancer (Kamerkar et al., 2017). An understanding of the regulatory mechanisms of miRNA and lncRNA that function upstream of *KRAS* in pancreatic cancer will be extremely valuable. Thus, the aim of this study was to study the regulatory mechanism of miRNA and lncRNA upstream of *KRAS* in exosomes derived from pancreatic cancer MSCs.

## Materials and Methods

### Cell Culture

Human mesenchymal stem cells (hMSCs) and the human pancreatic cancer cell line (CFPAC-1) were purchased from the Shanghai cell bank of the Chinese Academy of Sciences. hMSCs were cultured in hMSC culture medium (Cyagen Biosciences Inc., Guangdong, China, # HUXMA-90011) with 10% fetal bovine serum (FBS) (Gibco, Invitrogen, Carlsbad, CA, United States, #10099-141) and 1% penicillin/streptomycin (#BS734, Sangon Biotech, Shanghai, China). CFPAC-1 cells were cultured in Iscove’s modified Dulbecco’s medium (IMDM, Gibco #12440053) supplemented with 10% FBS (Gibco #10099-141) and 1% penicillin/streptomycin (#BS734, Sangon Biotech, Shanghai, China).

### Exosome Purification

Cells growing in the logarithmic phase were digested using 1 mL of 0.25% pancreatic enzymes; digestion was terminated using 2 mL of complete medium. Cells were transferred into a 15 mL centrifuge tube (Corning, NY, United States, #430790) and centrifuged for 3 min at 1500 rpm. Cell numbers were counted using a cell counting plate. Cells were seeded in a 100 mm culture dish (Corning #430167) at a density of 80% after cell apposition and incubated at 37°C overnight in an atmosphere of 5% CO_2_. Following cell apposition, the culture medium was replaced with a medium containing 10% exosome-free serum and subsequently further incubated for 48 h. The supernatant was collected in a 50 mL centrifuge tube (Corning #430828) and centrifuged at 300 × *g* for 10 min, followed by 2000 × *g* for 20 min to remove the cells, and further at l0000 × *g* for 30 min to remove the subcellular component. A final centrifugation was performed at l0000 × *g* for 60 min to obtain the exosomes. The obtained exosomes were suspended in 30 mL phosphate-buffered saline (PBS) solution, followed by centrifugation at 10,0000 × *g* 60 min. The purified exosomes were suspended in 1 mL PBS solution and stored at 4°C for further experiments.

### Transmission Electron Microscopy (TEM) and Malvern Particle Size Analysis

Purified exosomes (20 μL) were diluted with 80 μL PBS, and 100 μL 5% glutaraldehyde was added to obtain a final concentration of 2.5% glutaraldehyde. Following overnight fixation at 4°C, exosomes were observed using a transmission electron microscopy (TEM). Purified exosomes (10 μL) were diluted with 990 μL PBS and exosome size was subsequently detected using a Malvern NS300 laser particle size analyzer.

### Western Blot Analysis

Collected exosomes were lysed and incubated on ice for 30 min. Thereafter, they were vortexed every 10 min to fragment the samples. Following centrifugation at 10000–14000 *g* for 10 min at 4°C, the supernatant was collected and stored at −80°C. The absorbance of the sample was read at 562 nm using an enzyme-labeled meter (TECAN, Infinite M100 PRO). Proteins were separated using sodium dodecyl sulfate-polyacrylamide gel electrophoresis (SDS-PAGE), and subsequently transferred to polyvinylidene fluoride (PVDF) (Millipore, MA, United States, #IPVH00010) membranes. The membranes were probed overnight with the primary antibody (CD63, rabbit polyclonal antibody; 26 kDa; 1:1000 dilution with 5% non-fat milk; Proteintech Group, Chicago, IL, United States; #25682-1-Ig) at 4°C. Thereafter, the membranes were probed with the secondary antibody (Peroxidase AffiniPure Goat Anti-Rabbit IgG; 1:10000 dilution; Jackson; #111-035-045) for 2 h at 37°C. Chemiluminescence corresponding to proteins was visualized using the Millipore ECL system (Tanon 4600, Shanghai, China).

### Quantitative Real-Time PCR (qRT-PCR) Analysis

Total RNA of exosomes was obtained using TRIzol reagent (TaKaRa Biotechnology, Shiga, Japan; #9109) according to the manufacturer’s instructions. Based on our bioinformatic prediction and literature research, the expression of 12 miRNAs (hsa-miR-494-3p, hsa-miR-212-3p, hsa-miR-142-3p, hsa-miR-203-3p, hsa-miR-30a-3p, hsa-miR-193a-3p, hsa-miR-3148, hsa-miR-4314, hsa-miR-217-5p, hsa-miR-330-5p, hsa-miR-143-3p, and hsa-miR-30d-3p) potentially related to *KRAS* and pancreatic cancer was detected by quantitative real-time PCR (qRT-PCR). First, cDNA was synthesized and the analysis performed using the PrimeScript RT Reagent Kit (TaKaRa, Shiga, Japan; #RR036A) in a system containing 1 μg total RNA, 1 μL RT primer, 4 μL 5× PrimeScript RT Master MIX (perfect Real Time), and the standard protocol of the Applied Biosystems 7500 Fast Real-Time PCR System (ABI, Foster City, CA, United States). Thereafter, a total of 4.6 μL cDNA was used as a template for PCR (10 μL) using SYBR Premix EX Taq (2×) (TaKaRa, #RR420A). The following were the reaction conditions used for PCR: 50.0°C for 3 min, 95.0°C for 3 min, followed by 40 cycles of 95.0°C for 10 s and 60.0°C for 30 s. The internal control for exosomes was consistent, and largely dependent on the type of sample being investigated. Thus, U6 or the above miRNAs that were stably expressed in both cells were selected as internal controls. All primers used in this study are summarized in Supplementary Table 1; each experiment was repeated three times. The relative expression of genes was calculated using the 2^–^^ΔΔ*Ct*^ method.

### Functional Verification of Hsa-miR-143-3p

#### Hsa-miR-143-3p Inhibitor Transfection

Hsa-miRA-143-3p inhibitor (5′-GAGCUACAGUGCUUCA UCUCA-3′) and hsa-miRA-143-3p inhibitor NC (5′-CAGUACUUUUGUGUAGUACAA-3′) were synthesized (Shanghai GenePharma Co., Ltd., Shanghai, China) and transfected into the hMSCs using Lipofectamine 2000 (Invitrogen life Technologies, #11668-027) according to the manufacturer’s instructions. Cells were harvested 48 h post-transfection for purifying the exosomes. The liquid supernatant was collected in a 50 mL centrifuge tube, and the sample was centrifuged at 300 × *g* for 10 min and 2000 × *g* for 20 min. After the sediment was discarded and cells removed, centrifugation was performed at 8,000 × *g* for 30 min to discard the sediment once again and remove the subcellular component. Thereafter, centrifugation was performed at 4°C, 8,000 × *g* for 30 min, and the liquid supernatant was concentrated to <10 mL. The concentrated medium was incubated overnight with the exosome separation solution (1:1), at 4°C. Finally, exosomes were obtained after centrifugation at 8,000 × *g* for 30 min and stored at 4°C for subsequent analysis.

#### qPCR Analysis

RNA (hsa-miR-143-3p) was isolated from exosomes by adding 900 μL TRIzol to 100 μL exosomes. qPCR was performed as mentioned previously; the sequence of the hsa-miR-143-3p primer is provided in Supplementary Table 1.

#### Cell Proliferation, Cell Apoptosis, Cell Cycle, Migration, and Invasion Analyses

The Cell Counting Kit-8 (CCK-8) (Beyotime, China #C0039) assay was used to measure cell proliferation/viability according to the manufacturer’s instructions. Cells were inoculated in 96-well plates at 4000 cells/well and cultured at 37°C overnight with 5% CO_2_. Following cell apposition, cells were divided into the following three groups: CFPAC-1only without MSC exosome (MOCK) group, CFPAC-1 + MSC-hsa-miR-143-3p inhibitor negative control (inhibitor NC) group, and CFPAC-1 + MSC- hsa-miR-143-3p inhibitor (Inhibitor) group. There were four concentrations for exosomes: 50, 100, 200, and 400 ng/μL for 24, 48, and 72 h. Based on the results for cell viability at four concentrations in each group, a final exosomes concentration was determined for the following experiments.

Cell cycle distribution and apoptosis in these three groups were analyzed after a 48 h incubation using flow cytometry. CFPAC-1 cells were treated with exosomes (200 ng/μL) and cultured for 48 h. Cell Cycle and Apoptosis Analysis Kit (BD Biosciences, United States #40301ES50) was used according to the manufacturer’s instructions. Cell apoptosis was detected using Annexin V-FITC/propidium iodide (PI). Cells were harvested and fixed with ethanol (70%) under 4°C. Then cells were stained with RNase and propidium iodide at 37°C without light for half an hour, and then tested by flow cytometer (BD Biosciences; NJ, United States) for cell cycle analysis.

Cell migration and invasion experiments were conducted in all three groups after a 48 h incubation using transwell dishes. Matrigel (BD Biosciences, United States #354234) was diluted with 1:20 serum-free medium and seeded in a Transwell chamber with 100 μL/well. CFPAC-1 cells (3 × 10^4^ cells/well) were seeded to the chamber with 100 μl of serum-free DMEM and then incubated with or without exosomes (200 ng/μL) at 37°C for 48 h. Then 4% paraformaldehyde was used to fix the invaded cells for 30 min, followed by crystal violet staining for 2 h. Finally, the cells were counted using a light microscope.

### qPCR and Western Blot Analyses for Genes and lncRNAs Related to miR-143

To investigate the associated mechanisms of hsa-miR-143-3p in MSC exosomes of pancreatic cancer, several upstream lncRNAs and genes downstream of hsa-miR-143-3p were screened using qPCR and western blot analyses. Firstly, the MicroRNA-lncRNA Targets module of the online mirwalk2.0 database^1^ was utilized for lncRNA prediction under the default setting parameter as the selected start position of miRNA seed: Position 1 had a Minimum seed length of 7 and *p*-value of 0.05. Thereafter, based on our prediction result and research, four lncRNAs [MALAT1 (EnsTransID: ENST00000534336), DGCR5 (EnsTransID: ENST00000440005, SNHG1 (EnsTransID: ENST00000538654), and HOTTIP (EnsTransID: ENST00000472494)] reported to be associated with pancreatic cancer and one lncRNA [RP11-363N22.3 (EnsTransID: ENST00000381466)] with the highest seed hsa-miR-143-3p sequence length were selected (Supplementary Excel 1). The binding sites of these lncRNAs and hsa-miR-143-3p were obtained using miRanda v3.3a software (Supplementary Table 3). In addition, eight genes (KrasG12D, PI3K, Akt, ERK, JNK, p38 MAPK, E-cadherin, and vimentin) that had been reported as targets of hsa-miR-143-3p in other cancers were focused on in our study (Tavanafar et al., 2017; Akao et al., 2018).

qPCR and western blot analyses were conducted for the three groups (MOCK, inhibitor NC, and inhibitor groups) after 48 h of cell culture according to previous procedures. The expression of five lncRNAs and eight genes was determined; primer sequences are summarized in Supplementary Table 2. GAPDH was used as an internal control. qPCR was performed as mentioned previously. In addition, the expression of proteins AKT, p-AKT, ERK2, p-ERK2, and Ras (G12D) was determined in all three groups. β-actin was used as an internal control. Western blot analysis was performed as mentioned previously. The membranes were probed overnight with primary rabbit antibodies (1:1000 dilution with 5% non-fat milk) at 4°C: AKT (Proteintech, #10176-2-AP), p-AKT (Proteintech, #66444-1-Ig), ERK2 (Wanleibio, #WL01864), p-ERK2 (Wanleibio, Technology Co., LTD., Shenyang, China, #WLP1512), Ras (G12D Mutant Specific, Cell Signaling Technology, MA, United States, #14429), and β-actin (Cell Signaling Technology, #4970). Thereafter, membranes were probed with secondary antibodies (rabbit antibody, 1:10000 dilution) for 2 h at 37°C.

### Verification of miRNA-143-3p Targeted lncRNA RP11-363N22.3

Among the predicted lncRNAs, lncRNA RP11-363N22.3 (EnsTransID: ENST00000381466) had not been investigated in pancreatic cancer and had the highest combination possibility of hsa-miR-143-3p among predicted lncRNAs. Thus, we focused on the relationships between hsa-miR-143-3p and lncRNA RP11-363N22.3. The binding sites of hsa-miR-143-3p and lncRNA RP11-363N22.3 were as follows:

Query: 3′ cuCGAUGUC–ACG—–AAGUAGAGu 5′
| : | | | | | | | | | | | | | | | |
ef: 5′ aaGTTACAGTTTGCACAAGTTCATCTCa 3′

The upstream and downstream 100 bp of the binding site were intercepted for sequence synthesis. The sequences were as follows: ENST00000381466-WT:

AGGAAAATGTTTTTTCCTACTATTGTTATAAAATAGTT TGGGCCACTTCTACTTCCTAGTATCCCTGGATTACGTAAC AGTAACAGCTGCGATTCAGTTCCACATTCTACATGTAAG GAGCGAGGGGTGGGAGCCCAGCCTTCCTGCCTTGCATC TCCC**CTGTCCTTGTGCTTCATCTCA**GCAGCATTACGTCA TACTTGCTAATAATTCACCTAAGAGTGACAAAAATAATTT TTATAAGCATGTCACAAAAAGGAAGGAAAAGTTTCTTTT CCCTGCTTTGATATTAGAACCAAATGGAGCTCTGGAAGT TTTCAATAAAATGAAAAATGATTT

ENST00000381466-MUT:

AGGAAAATGTTTTTTCCTACTATTGTTATAAAATAGTT TGGGCCACTTCTACTTCCTAGTATCCCTGGATTACGTAAC AGTAACAGCTGCGATTCAGTTCCACATTCTACATGTAAG GAGCGAGGGGTGGGAGCCCAGCCTTCCTGCCTTGCATC TCCC**CTGTCCTTACATCCTGCTCTA**GCAGCATTACGTCA TACTTGCTAATAATTCACCTAAGAGTGACAAAAATAATTT TTATAAGCATGTCACAAAAAGGAAGGAAAAGTTTCTTT TCCCTGCTTTGATATTAGAACCAAATGGAGCTCTGGAAG TTTTCAATAAAATGAAAAATGATTT (The sequence region in bold represents the wild type and the mutated binding sites, respectively). This sequence was constructed to match the *Nhe*I/*Xba*I cleavage sites of the pMIR-Glo vector. Thereafter, transfection was performed according to the standard protocol. A total of six groups were transfected as follows: (1) pMIR-Glo plasmid 200 ng + hsa-miR-143-3p-NC 0.5 μL; (2) pMIR-Glo plasmid 200 ng + hsa-miR-143-3p mimic 0.5 μL; (3) pMIR-Glo–LncRNA RP11-363N22.3 WT plasmid 200 ng + hsa-miR-143-3p-NC 0.5 μL; (4) pMIR-Glo–LncRNA RP11-363N22.3 WT plasmid 200 ng + hsa-miR-143-3p mimic 0.5 μL; (5) pMIR-Glo–LncRNA RP11-363N22.3 MUT plasmid 200 ng + hsa-miR-143-3p-NC 0.5 μL; and (6) pMIR-Glo–LncRNA RP11-363N22.3 MUT plasmid 200 ng + hsa-miR-143-3p mimic 0.5 μL. The dual-luciferase reporter gene system (Promega #e1910) was used for gene detection.

### Tumorigenesis Test in Nude Mice

Fifteen Balb/c nude mice (4–6 weeks old) were randomly divided into three groups (MOCK, CFPAC-1 + MSC-hsa-miR-143-3p mimics NC (mimics NC) group, and CFPAC-1 + MSC-hsa-miR-143-3p mimics (mimics) group, with five mice per group). Each mouse was injected subcutaneously with 100 μL of CFPAC-1 cell suspension in the subaxillary of the right forelimb. Tumor diameters were measured 2 weeks post-injection. Mice in the MOCK group received an injection of 50 μL PBS, mice in the mimic NC group received an injection of MSC cells (10^6^ cells resuspended in 50 μL PBS) transfected with hsa-miR-143-3p NC, and mice in the mimic group received an injection of MSC cells (10^6^ cells resuspended in 50 μL PBS) transfected with hsa-miR-143-3p mimic in tumor.

After a period, tumorigenesis was monitored and tumor volumes were calculated three times a week (volume = length × width^2^/2). Thereafter, the tumors were excised, fixed, and subsequently processed in paraffin wax. Approximately 4–7 μm thick sections were made and stained with hematoxylin and eosin (H&E, Sigma #H9627). Dry sections were observed under a microscope and photographed.

### Statistical Analysis

All results were represented as the mean ± SD. Each experiment was repeated three times. Data were analyzed using GraphPad Prism (GraphPad Software, San Diego, CA, United States) software. Differences between two groups were measured using the Student’s *t* test and multiple comparisons were performed by Newman-Keuls Multiple Comparison Test. *P* < 0.05 was regarded as a statistically significant difference.

## Results

### TEM, Malvern Particle Size Analysis, and Western Blot Verification of Exosomes

**FIGURE 1:**
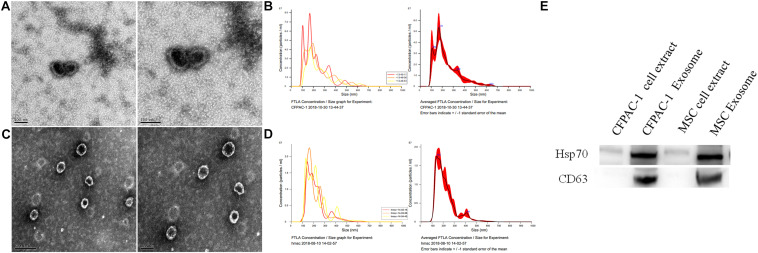
Exosome detection. Transmission electron microscope (TEM) analysis **(A)** and Malvern particle size detection **(B)** for exosomes from human pancreatic cancer cell line (CFPAC-1); TEM analysis **(C)** and Malvern particle size detection **(D)** for human mesenchymal stem cells (hMSCs). Detection of CFPAC-1- and hMSC-derived exosomes markers by western blot analysis **(E)**.

As shown in Figure 1, the microscopy and particle size analyses of exosomes from CFPAC-1 and hMSCs confirmed the identity of exosomes based on their appearance and size. Moreover, CD63 and HSP70 are regarded as detection markers for exosomes, which have been found in both CFPAC-1 and hMSC exosomes (Figure 1D).

### Differential Expression of miRNAs Between CFPAC-1 and hMSC Exosomes

**FIGURE 2:**
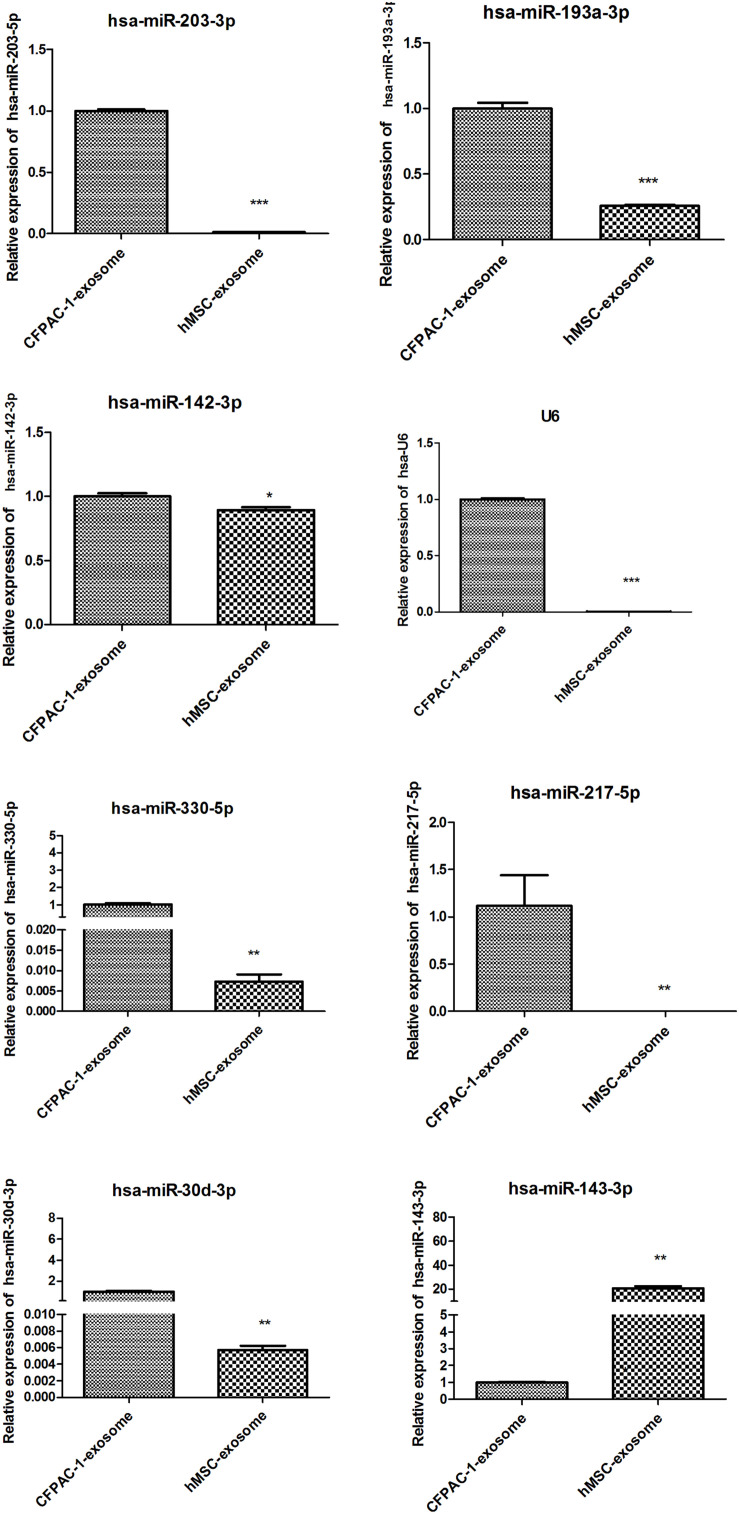
Detection of the relative expression of eight miRNAs by quantitative polymerase chain reaction (qPCR). *p < 0.05; **p < 0.01; and ***p < 0.001. hsa-miR-494-3p was stably expressed without significant difference in both CFPAC-1 and hMSC exosomes (*p* > 0.05), thus hsa-miR-494-3p was selected as the internal control.

Based on our bioinformatic prediction and research, 12 miRNAs were shortlisted and analyzed using qPCR analysis. The results showed that U6 expression between CFPAC-1 and hMSC exosomes was significantly different (*p* < 0.001), while hsa-miR-494-3p was stably expressed without significant differences in both CFPAC-1 and hMSC exosomes (*p* > 0.05), thus hsa-miR-494-3p was selected as the internal control (data not shown). There was a significant decrease in the expression of hsa-miR-203-3p, hsa-miR-193a-3p, hsa-miR-142-3p, hsa-miR-330-5p, hsa-miR-217-5p, and hsa-miR-30d-3p (*p* < 0.05), whereas the expression of hsa-miR-212-3p, hsa-miR-30a-3p, hsa-miR-3148, and hsa-miR-4314 were not detected in CFPAC-1 and hMSC exosomes. hsa-miR-143-3p expression markedly increased in hMSC exosomes (*p* < 0.01, Figure 2), which was selected for subsequent analysis.

### Functional Verification of hsa-miR-143-3p

#### The Effect of hsa-miR-143-3p in Exosome on Cell Proliferation

**FIGURE 3:**
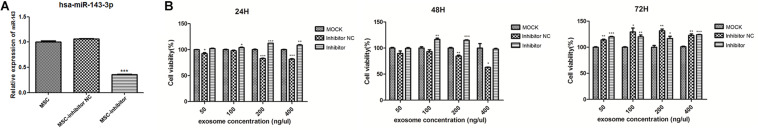
Verification data for the hsa-miR-143 inhibitor. **(A)** Quantitative polymerase chain reaction (qPCR) results of hsa-miR143-3P; **(B)** Results of Cell Counting Kit-8 (CCK-8) experiments at 24, 48, and 72 h. **p < 0.01 and ***p < 0.001 compare to the MOCK group. MOCK: CFPAC-1 only (without MSC exosome); Inhibitor NC: CFPAC-1 + MSC-hsa-miR-143-3p inhibitor negative control; Inhibitor: CFPAC-1 + MSC-miR-143 inhibitor group.

qPCR results showed that hsa-miR143-3p expression significantly decreased in the inhibitor group compared to the MOCK and NC groups (*p* < 0.001; Figure 3A). Results of the CCK-8 assay at 24 h showed that cell viability of the NC group was markedly lower than that of the MOCK group at concentrations of 50, 200, and 400 ng/μL, and cell viability of the inhibitor group was markedly higher than that of the MOCK group at the same concentrations. At 48 h, cell viability of the NC group was markedly lower than that of the MOCK group at concentrations of 200 and 400 ng/μL, and cell viability of the inhibitor group was markedly higher than that of the MOCK group at concentrations of 100 and 200 ng/μL. At 72 h, cell viability of the inhibitor and inhibitor NC groups was markedly higher than that of the MOCK group. Therefore, the exosome concentration of 200 ng/μL and 48 h time point were selected to perform subsequent experiments (Figure 3B).

#### The Effect of Hsa-miR-143-3p in Exosomes on Cell Apoptosis, Cell Cycle, Cell Invasion, and Migration

**FIGURE 4:**
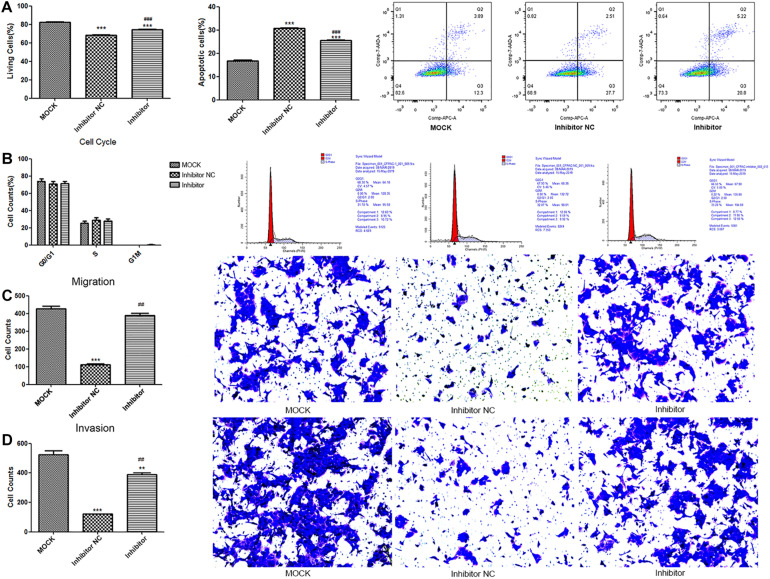
The results for the determination of cytological effect in the MOCK, inhibitor NC, and inhibitor groups. The quantitative statistics and representative images of cell apoptosis **(A)** and cell cycle **(B)** determined by flow cytometer; The quantitative statistics and representative images of crystal violet stained migrated cells **(C)** and invaded cells **(D)** determined by Transwell assay. Cell apoptosis was detected by **p < 0.01 and ***p < 0.001 compare to the MOCK group; ^##^*p* < 0.01 and ^###^*p* < 0.001 compared to NC group. MOCK: CFPAC-1 only (without MSC exosome); Inhibitor NC: CFPAC-1 + MSC-hsa-miR-143-3p inhibitor negative control; Inhibitor: CFPAC-1 + MSC-miR-143 inhibitor group.

Compared to the MOCK group, the live cell count was significantly reduced and apoptotic cells significantly increased in the inhibitor and NC groups (*p* < 0.001). The live cell count in the inhibitor group was significantly higher, and the apoptotic cells were significantly lower than those in the NC group (*p* < 0.001, Figure 4A). As illustrated in Figure 4B, no significant difference was observed for cell cycle among the groups. Furthermore, cell migration and invasion were significantly reduced in the NC group (*p* < 0.001) in comparison to the MOCK group. Alternatively, the cell invasion and migration ability significantly increased in the inhibitor group compared to that in the NC group (*p* < 0.01; Figures 4C,D).

### qPCR Detection and Western Blot Analysis for Genes and lncRNAs Related to miR-143

**FIGURE 5:**
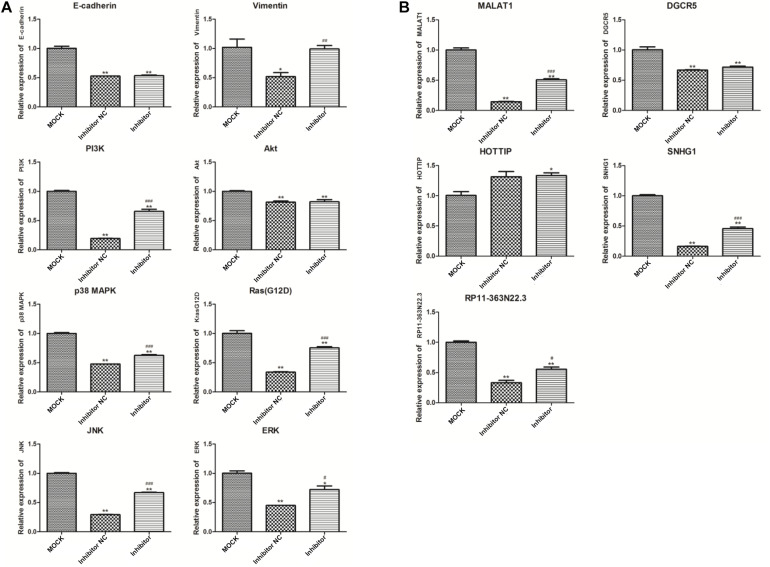
Expression detection of eight genes **(A)** and five lncRNAs **(B)** by quantitative polymerase chain reaction (qPCR). *p < 0.05 and **p < 0.01 compared to the MOCK group; ^#^*p* < 0.05, ^##^*p* < 0.01, and ^###^*p* < 0.001 compared to the NC group. GAPDH was selected as the internal control. MOCK: CFPAC-1 only (without MSC exosome); Inhibitor NC: CFPAC-1 + MSC-hsa-miR-143-3p inhibitor negative control; Inhibitor: CFPAC-1 + MSC-miR-143 inhibitor group.

As shown in Figure 5, the expression of five lncRNAs and eight genes were screened in this study. Among these lncRNAs, expression of MALAT1, SNHG1, and RP11-363N22.3 in the NC group significantly decreased compared to that in the MOCK group (*p* < 0.01), but increased in the inhibitor group compared to that in the NC group (*p* < 0.05 or *p* < 0.01). In addition, no significant change of DGCR5 and HOTTIP expressions were measured between inhibitor and NC groups. Furthermore, expression of the six genes, KrasG12D, PI3K, ERK, JNK, p38 MAPK, and vimentin, were reduced in the NC group compared to that in the MOCK group, and increased in the inhibitor group compared to that in the NC group (all, *p* < 0.05).

**FIGURE 6:**
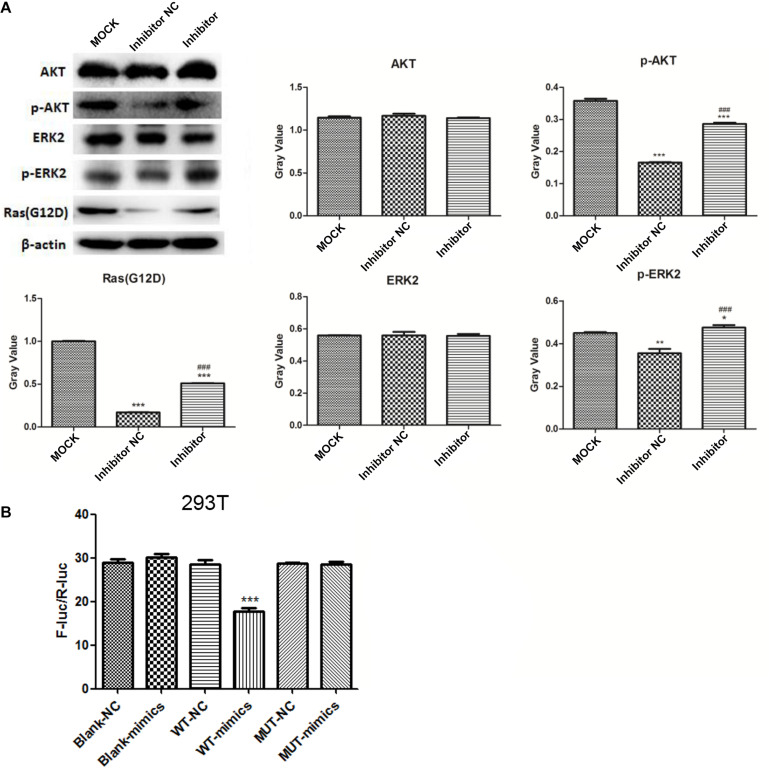
Western blot analysis of AKT, ERK2, p-AKT, p-ERK2, and RasG12D **(A)** *p < 0.05, **p < 0.01, and ***p < 0.001 compared to the MOCK group; ^###^*p* < 0.001 compared to the NC group. Dual-luciferase reporter gene assay results for the targeted combinatorial effect between the lncRNA RP11-363N22.3s and hsa-miR-143-3p **(B)**. ***p < 0.001 compared to the NC group. MOCK: CFPAC-1 only (without MSC exosome); Inhibitor NC: CFPAC-1 + MSC-hsa-miR-143-3p inhibitor negative control; Inhibitor: CFPAC-1 + MSC-miR-143 inhibitor group.

Western blot analysis (Figure 6A) showed no significant difference in the expression of AKT and ERK2 among the groups. Expression of p-AKT, p-ERK2, and RasG12D significantly decreased in the NC group compared to that in the MOCK group (both, *p* < 0.05), and markedly increased in the inhibitor group compared to that in the NC group (*p* < 0.001).

### Dual-Luciferase Reporter Gene Assay

The results of the dual-luciferase reporter gene assay showed a significant difference between WT-mimic and WT-NC (*p* < 0.001), suggesting a targeted combinatorial effect between lncRNA RP11-363N22.3 and hsa-miR-143-3p (Figure 6B).

### Tumorigenesis Test in Nude Mice

**FIGURE 7:**
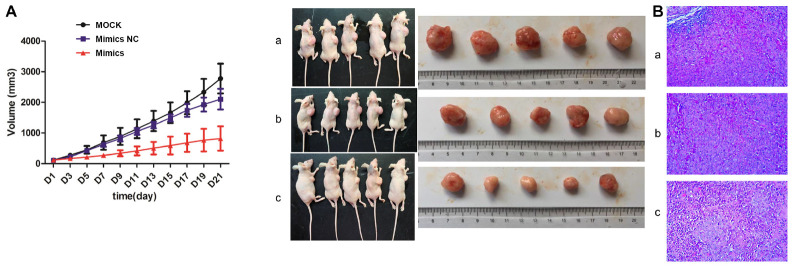
Tumorigenesis results in nude mice. **(A)** Tumor volume; **(B)** Hematoxylin and eosin (H&E) staining. a: MOCK group; b: Mimics NC group; c: Mimics group. MOCK: CFPAC-1 only (without MSC exosome); Mimics NC: CFPAC-1 + MSC-hsa-miR-143-3p mimics NC; Mimics: CFPAC-1 + MSC-hsa-miR-143-3p mimics. Mice in the MOCK group received an injection of 50 μL PBS, mice in mimics NC group received an injection of MSC cells (10^6^ cells resuspended in 50 μL PBS) transfected with hsa-miR-143-3p NC, and mice in mimics group received an injection of MSC cells (10^6^ cells resuspended in 50 μL PBS) transfected with hsa-miR-143-3p mimic in tumor.

As shown in Figure 7, the tumor volume of the mimics NC group was smaller than that of the MOCK group, and the tumor volume of the mimics group was smaller than that of the mimics NC group. The overall trend of tumor volume was MOCK group > mimics NC group > mimics group. H&E staining showed that there were no obvious pathological changes in MOCK and mimic NC groups, while cell necrosis was seen in some regions in the mimics group.

## Discussion

Pancreatic cancer is a highly aggressive gastrointestinal cancer with extremely poor prognosis (Wei et al., 2018). In the present study, hsa-miR-143-3p expression significantly increased in hMSC exosomes compared to that in CFPAC-1 exosomes (*p* < 0.01). The qPCR results confirmed that hsa-miR143-3P expression significantly decreased in the inhibitor group compared to the NC and MOCK groups (*p* < 0.001). CCK-8 assay results showed that the exosome concentration of 200 ng/μL in 48 h was suitable for performing subsequent experiments. The number of apoptotic cells and cell invasion and migration assays showed significant changes in the inhibitor group compared to the inhibitor NC and MOCK groups. However, there was no significant difference in the cell cycle among the groups. In addition, the expression of three lncRNAs (MALAT1, SNHG1, and RP11-363N22.3) and six genes (KrasG12D, PI3K, ERK, JNK, p38 MAPK, and vimentin) significantly decreased in the NC group compared to that in the MOCK group and increased in the inhibitor group compared to that in the NC group. The results of the dual-luciferase reporter gene assay suggested a targeted combinatorial effect between lncRNA RP11-363N22.3 and miR-143. The tumorigenesis test in nude mice demonstrated that the overall trend of tumor volume was MOCK group > mimic NC group > mimic group. H&E staining results revealed that there were no obvious pathological changes in MOCK and mimic NC groups, while cell necrosis was observed in some regions in the mimic group.

Our current data showed that the expression of hsa-miR-143-3p significantly increased in hMSC exosomes compared to that in CFPAC-1 exosomes, suggesting the existence of hsa-miR-143-3p in exosomes derived from hMSC. Consistently, Che et al. (2019) showed hsa-miR-143 was overexpressed in MSCs-derived exosomes in prostate cancer. The qPCR results verified that hsa-miR143-3P expression markedly decreased in the inhibitor group compared with the NC and MOCK groups (*p* < 0.0001). Studies have shown that miR-143 was down-regulated in pancreatic cancer and was critical for cancer development (Pham et al., 2013; Kamerkar et al., 2017).

The expression of three lncRNAs (MALAT1, SNHG1, and RP11-363N22.3) and six genes (KrasG12D, PI3K, ERK, JNK, p38MAPK, and vimentin) significantly decreased in the NC group compared to that in the MOCK group, and increased in the inhibitor group compared to that in the NC group, suggesting that KrasG12D, PI3K, ERK, JNK, p38MAPK, and vimentin were targets of hsa-miR-143-3p and that a targeted combinatorial effect exists between the three lncRNAs (MALAT1, SNHG1, and RP11-363N22.3) and hsa-miR-143-3p. Previous studies showed that KrasG12D was associated with pancreatic cancer (Sodir et al., 2015). Tavanafar et al. (2017) suggested that the Akt/PI3K signaling pathway may be a therapeutic strategy for pancreatic cancer treatment. Sun et al. (2016) suggested that the Akt/PI3K signaling played a role in angiogenesis of pancreatic cancer. Liu et al. (2015) indicated that the Akt/ERK pathways were involved in cell proliferation and apoptosis in pancreatic cancer. The ERK/MAPK pathway can inhibit cellular reprogramming and tumorigenesis of pancreatic cancer (Calvé et al., 2015). Taniuchi et al. (2014) suggested that p38MAPK activity affected cell invasion in pancreatic cancer. Previous studies showed that JNK played a role in the invasion of pancreatic cancer cells (Yuan et al., 2015; Cai et al., 2017). Zhu et al. (2016) suggested that vimentin was involved in the tumorigenesis of pancreatic cancer. In addition, Pang et al. (2015) indicated that the lncRNA, MALAT1, may be a biomarker for the prognosis of patients with pancreatic cancer. Jiao et al. (2014) showed that the lncRNA, MALAT1, may be involved in the malignant phenotypes of pancreatic cancer and may thus be regarded as a therapeutic target. It was reported that the lncRNA, SNHG1, was involved in cell proliferation in gastric and cervical cancers, among others (Ekstrand et al., 2004; Que et al., 2018). However, we did not find any previous reports about the role of the lncRNA, RP11-363N22.3, in pancreatic or other cancers. Based on our current data and previous studies, we hypothesize that KrasG12D, PI3K, ERK, JNK, p38MAPK, vimentin, lncRNA MALAT1, lncRNA SNHG1, and lncRNA RP11-363N22.3 targeted by hsa-miR-143-3p may play crucial roles in pancreatic cancer.

Furthermore, in the present study, compared to the NC and MOCK groups, the number of apoptotic cells and levels of cell invasion and migration were significantly different in the inhibitor group; however no significant difference was observed for cell cycles among the groups. In addition, the overall trend of tumor volume was MOCK group > mimics NC group > mimic group. These results suggest that hsa-miR-143-3p promotes cell apoptosis and suppresses cell growth, invasion, and migration but does not affect the cell cycle in CFPAC-1, which is in agreement with previous studies (Lei et al., 2017; Chen and Xiaowen, 2018), where hsa-miR-143-3p was involved in cell apoptosis, cell invasion, and proliferation in cancers.

In summary, hsa-miR-143-3p may regulate KrasG12D, PI3K, ERK, JNK, p38MAPK, and vimentin synergistically to promote apoptosis and suppress cell growth, invasion, and migration in pancreatic cancer. Furthermore, lncRNAs MALAT1, SNHG1, and RP11-363N22.3 may also play a role in pancreatic cancer via hsa-miR-143-3p. However, further studies are necessary to determine the regulatory mechanism of other miRNAs or lncRNAs that play a crucial role in pancreatic cancer. The effects of hsa-miR-143-3p studied by mimic transfection with respect to apoptosis, cell cycle, cell invasion, migration, RT-PCR, and western blot analyses require further for verification.

## Data Availability Statement

The original contributions presented in the study are included in the article/Supplementary Material, further inquiries can be directed to the corresponding author/s.

## Ethics Statement

The animal study was reviewed and approved by the Ethics Committee of Shanghai Tongji Hospital.

## Author Contributions

BW and BS conceived and designed the project. YX and BW collected the data. YW, LL, and NL interpreted the data and performed the statistical analysis. XW wrote the manuscript. RL revised the manuscript. All authors read and approved the final manuscript.

## Conflict of Interest

The authors declare that the research was conducted in the absence of any commercial or financial relationships that could be construed as a potential conflict of interest.

## Footnotes

1 http://zmf.umm.uni-heidelberg.de/apps/zmf/mirwalk2/

## Supplementary Material

Supplementary Table 1 The primers used in the Quantitative real-time PCR (qRT-PCR).

Supplementary Table 2 The primer sequence of five lncRNA and eight genes.

Supplementary Table 3 Binding sites of five detected lncRNAs and miR-143-3p.

Supplementary Excel 1 Predicted upstream lncRNAs of miR-143-3p based on bioinformatic analysis.
