# Supplementary material for: Human Mesenchymal Stem Cell-Derived Exosomal microRNA-143 Promotes Apoptosis and Suppresses Cell Growth in Pancreatic Cancer via Target Gene Regulation
Source: Front Genet. 2021 Feb 12;12:581694. doi: 10.3389/fgene.2021.581694 (PMC7907650; doi:10.3389/fgene.2021.581694)
Supplement: Supplementary Table 2 — The primer sequence of five lncRNA and eight genes. [file Table_2.DOCX]

**Table S1** The primers used in the Quantitative real-time PCR (qRT-PCR).

| Name | Primer sequence (5’-3’) |
| --- | --- |
| hsa-miR-494-RT | GTCGTATCCAGTGCAGGGTCCGAGGTATTCGCACTGGATACGACGAGGTT |
| JH-hsa-miR-494-F | GCGCTGAAACATACACGGGA |
| hsa-miR-142-3p-RT | GTCGTATCCAGTGCAGGGTCCGAGGTATTCGCACTGGATACGACTCCATA |
| JH-hsa-miR-142-3p-F | GCGCTGTAGTGTTTCCTACTT |
| hsa-miR-203-RT | GTCGTATCCAGTGCAGGGTCCGAGGTATTCGCACTGGATACGACCTAGTG |
| JH-hsa-miR-203-F | GCGCGTGAAATGTTTAGGAC |
| hsa-miR-193a-3p-RT | GTCGTATCCAGTGCAGGGTCCGAGGTATTCGCACTGGATACGACACTGGG |
| JH-hsa-miR-193a-3p-hF | GGCAACTGGCCTACAAAGT |
| hsa-miR-217-5p-RT | GTCGTATCCAGTGCAGGGTCCGAGGTATTCGCACTGGATACGACTCCAAT |
| JH-hsa-miR-217-5p-F | CGCTACTGCATCAGGAACTG |
| hsa-miR-330-5p-RT | GTCGTATCCAGTGCAGGGTCCGAGGTATTCGCACTGGATACGACGCCTAA |
| JH-hsa-miR-330-5p-F | GCTCTCTGGGCCTGTGTC |
| hsa-miR-143-3p-RT | GTCGTATCCAGTGCAGGGTC |
| JH-hsa-miR-143-3p-F | GCGCTGAGATGAAGCACTG |
| hsa-miR-30d-3p-RT | GTCGTATCCAGTGCAGGGTCCGAGGTATTCGCACTGGATACGACGCAGCA |
| JH-hsa-miR-30d-3p-F | GCGCCTTTCAGTCAGATGTT |
| U6-hF | CTCGCTTCGGCAGCACA |
| U6-hR | AACGCTTCACGAATTTGCGT |

**Table S2** The primer sequence of five lncRNA and eight genes.

| Name | Primer sequence (5’-3’) |
| --- | --- |
| MALAT1-hF | ATGCGAGTTGTTCTCCGTCTA |
| MALAT1-hR | CTGCCTCAATGCCTACCG |
| DGCR5-hF | GGAGAAGCGAACCAAGCC |
| DGCR5-hR | GCACCCACAGAAGCAGAGC |
| SNHG1-hF | ATTGAACTTCCCATAACTCCAC |
| SNHG1-hR | CAAATTGACAGCCAGTCCTC |
| HOTTIP-hF | GACCACAGGACGGACATCG |
| HOTTIP-hR | GGACAGGGAAGGGATAGGG |
| RP11-363N22.3-hF | CGGTTGCTCACGCCTCTA |
| RP11-363N22.3-hR | GGTTCAAGCGATTCTTCTGC |
| KrasG12D-hF | ACTTGTGGTAGTTGGAGCAGA |
| KrasG12D-hR | TTGGATCATATTCGTCCACAA |
| PI3K-hF | AGTAGGCAACCGTGAAGAAAAG |
| PI3K-hR | GAGGTGAATTGAGGTCCCTAAGA |
| Akt-hF | GTCATCGAACGCACCTTCCAT |
| Akt-hR | AGCTTCAGGTACTCAAACTCGT |
| ERK2-hF | TCACACAGGGTTCCTGACAGA |
| ERK2-hR | ATGCAGCCTACAGACCAAATATC |
| JNK-hF | TGTGTGGAATCAAGCACCTTC |
| JNK-hR | AGGCGTCATCATAAAACTCGTTC |
| p38 MAPK-hF | CTGTTGGACGTTTTTACACCTGC |
| p38 MAPK-hR | AGACCTCGGAGAATTTGGTAGA |
| E-cadherin-hF | CGAGAGCTACACGTTCACGG |
| E-cadherin-hR | GGGTGTCGAGGGAAAAATAGG |
| Vimentin-hF | TTGAACGCAAAGTGGAATC |
| Vimentin-hR | AGGTCAGGCTTGGAAACA |
| GAPDH-hF | TGACAACTTTGGTATCGTGGAAGG |
| GAPDH-hR | AGGCAGGGATGATGTTCTGGAGAG |

**Table S3** Binding sites of five detected lncRNAs and miR-143-3p.

1. **RP11-363N22.3-001**

Read Sequence:RP11-363N22.3-001 (1886 nt)

=-=-=-=-=-=-=-=-=-=-=-=-=-=-=-=-=-=-=-=-=-=-=-=-=-=-=-=-=-=-=-=

Performing Scan: hsa-miR-143-3p vs RP11-363N22.3-001

=-=-=-=-=-=-=-=-=-=-=-=-=-=-=-=-=-=-=-=-=-=-=-=-=-=-=-=-=-=-=-=

Forward: Score: 165.000000 Q:2 to 14 R:1710 to 1730 Align Len (12) (100.00%) (100.00%)

Query: 3' cucgauguCACGAAGUAGAGu 5'

||||||||||||

Ref: 5' ctgtccttGTGCTTCATCTCa 3'

Energy: -23.750000 kCal/Mol

Scores for this hit:

>hsa-miR-143-3p RP11-363N22.3-001 165.00 -23.75 2 14 1710 1730 12 100.00% 100.00%

Score for this Scan:

Seq1,Seq2,Tot Score,Tot Energy,Max Score,Max Energy,Strand,Len1,Len2,Positions

>>hsa-miR-143-3p RP11-363N22.3-001 165.00 -23.75 165.00 -23.75 1 21 1886 1710

1. **HOTTIP-005**

Read Sequence:HOTTIP-005 (2285 nt)

=-=-=-=-=-=-=-=-=-=-=-=-=-=-=-=-=-=-=-=-=-=-=-=-=-=-=-=-=-=-=-=

Performing Scan: hsa-miR-143-3p vs HOTTIP-005

=-=-=-=-=-=-=-=-=-=-=-=-=-=-=-=-=-=-=-=-=-=-=-=-=-=-=-=-=-=-=-=

Forward: Score: 132.000000 Q:3 to 17 R:2060 to 2080 Align Len (14) (71.43%) (78.57%)

Query: 3' cucgaUGUCACGAAGUAGAgu 5'

||| :|||||||

Ref: 5' gtgagACAAGATTTCATCTgt 3'

Energy: -12.330000 kCal/Mol

HOTTIP Scores for this hit:

>hsa-miR-143-3p HOTTIP-005 132.00 -12.33 3 17 2060 2080 14 71.43% 78.57%

Score for this Scan:

Seq1,Seq2,Tot Score,Tot Energy,Max Score,Max Energy,Strand,Len1,Len2,Positions

>>hsa-miR-143-3p HOTTIP-005 132.00 -12.33 132.00 -12.33 2 21 2285 2060

1. **SNHG1-003**

Read Sequence:SNHG1-003 (2768 nt)

=-=-=-=-=-=-=-=-=-=-=-=-=-=-=-=-=-=-=-=-=-=-=-=-=-=-=-=-=-=-=-=

Performing Scan: hsa-miR-143-3p vs SNHG1-003

=-=-=-=-=-=-=-=-=-=-=-=-=-=-=-=-=-=-=-=-=-=-=-=-=-=-=-=-=-=-=-=

Forward: Score: 137.000000 Q:2 to 15 R:841 to 862 Align Len (14) (78.57%) (85.71%)

Query: 3' cucgaugUCACGA-AGUAGAGu 5'

|||| | ||||||:

Ref: 5' actttcaAGTGATGTCATCTTa 3'

Energy: -17.600000 kCal/Mol

Scores for this hit:

>hsa-miR-143-3p SNHG1-003 137.00 -17.60 2 15 841 862 14 78.57% 85.71%

Forward: Score: 130.000000 Q:2 to 19 R:1446 to 1466 Align Len (17) (70.59%) (76.47%)

Query: 3' cucGAUGUCACGAAGUAGAGu 5'

| | ||| :|| |||||

Ref: 5' aacCCAAAGTCTTTGATCTCc 3'

Energy: -10.260000 kCal/Mol

Scores for this hit:

>hsa-miR-143-3p SNHG1-003 130.00 -10.26 2 19 1446 1466 17 70.59% 76.47%

Score for this Scan:

Seq1,Seq2,Tot Score,Tot Energy,Max Score,Max Energy,Strand,Len1,Len2,Positions

>>hsa-miR-143-3p SNHG1-003 267.00 -27.86 137.00 -17.60 5 21 2768 841 1446

1. **DGCR5-001**

Read Sequence:DGCR5-001 (1299 nt)

=-=-=-=-=-=-=-=-=-=-=-=-=-=-=-=-=-=-=-=-=-=-=-=-=-=-=-=-=-=-=-=

Performing Scan: hsa-miR-143-3p vs DGCR5-001

=-=-=-=-=-=-=-=-=-=-=-=-=-=-=-=-=-=-=-=-=-=-=-=-=-=-=-=-=-=-=-=

Forward: Score: 150.000000 Q:2 to 19 R:1167 to 1187 Align Len (17) (64.71%) (76.47%)

Query: 3' cucGAUGUCACGAAGUAGAGu 5'

| :||| : |||||||

Ref: 5' agcCAGCAGCTTCTCATCTCt 3'

Energy: -15.290000 kCal/Mol

Scores for this hit:

>hsa-miR-143-3p DGCR5-001 150.00 -15.29 2 19 1167 1187 17 64.71% 76.47%

Forward: Score: 143.000000 Q:2 to 20 R:221 to 241 Align Len (18) (77.78%) (83.33%)

Query: 3' cuCGAUGUCACGAAGUAGAGu 5'

|||: ||| || ||||||

Ref: 5' gaGCTGGAGTCCTCCATCTCc 3'

Energy: -23.290001 kCal/Mol

Scores for this hit:

>hsa-miR-143-3p DGCR5-001 143.00 -23.29 2 20 221 241 18 77.78% 83.33%

Score for this Scan:

Seq1,Seq2,Tot Score,Tot Energy,Max Score,Max Energy,Strand,Len1,Len2,Positions

>>hsa-miR-143-3p DGCR5-001 293.00 -38.58 150.00 -23.29 20 21 1299 1167 221

**5) MALAT1-001**

Read Sequence:MALAT1-001 (8708 nt)

=-=-=-=-=-=-=-=-=-=-=-=-=-=-=-=-=-=-=-=-=-=-=-=-=-=-=-=-=-=-=-=

Performing Scan: hsa-miR-143-3p vs MALAT1-001

=-=-=-=-=-=-=-=-=-=-=-=-=-=-=-=-=-=-=-=-=-=-=-=-=-=-=-=-=-=-=-=

Forward: Score: 147.000000 Q:2 to 17 R:5374 to 5395 Align Len (16) (81.25%) (87.50%)

Query: 3' cucgaUGUC-ACGAAGUAGAGu 5'

| || ||||||||:||

Ref: 5' tcagaAGAGTTGCTTCATTTCa 3'

Energy: -19.500000 kCal/Mol

Scores for this hit:

>hsa-miR-143-3p MALAT1-001 147.00 -19.50 2 17 5374 5395 16 81.25% 87.50%

Forward: Score: 146.000000 Q:2 to 20 R:3976 to 3997 Align Len (19) (63.16%) (73.68%)

Query: 3' cuCGA-UGUCACGAAGUAGAGu 5'

|:| |:| | |||||||

Ref: 5' tgGTTCATATTCAGTCATCTCa 3'

Energy: -13.290000 kCal/Mol

Scores for this hit:

>hsa-miR-143-3p MALAT1-001 146.00 -13.29 2 20 3976 3997 19 63.16% 73.68%

Forward: Score: 131.000000 Q:2 to 20 R:6840 to 6858 Align Len (18) (66.67%) (77.78%)

Query: 3' cuCGAUGUCACGAAGUAGAGu 5'

||| : || |||||:||

Ref: 5' atGCT-TTTTG-TTCATTTCt 3'

Energy: -10.710000 kCal/Mol

Scores for this hit:

>hsa-miR-143-3p MALAT1-001 131.00 -10.71 2 20 6840 6858 18 66.67% 77.78%

Score for this Scan:

Seq1,Seq2,Tot Score,Tot Energy,Max Score,Max Energy,Strand,Len1,Len2,Positions

>>hsa-miR-143-3p MALAT1-001 424.00 -43.50 147.00 -19.50 21 21 8708 5374 3976 6840
